# Hereditary Neuroses in Children.—I

**Published:** 1897-05-15

**Authors:** 


### Modern Sociology.

### HEREDITARY NEUROSES IN CHILDREN.—I.

By neurosis we mean such abnormal condition of the nervous system that functionally it tends to disorderly action. In every such case there is doubtless some deviation from the normal in the constitution of the nerve elements; yet with our present means of research we are unable to point to a definite pathological lesion as the cause of the neurosis. Neuralgia, migraine, epilepsy, hysteria, neurasthenia, are examples of the forms of neurosis most commonly met with; and certain mental, and even moral, eccentricities may be traced to the same cause. Though manifested by very definite symptoms, these affections are not as a rule characterised by visible defects of nerve structure, though advances in microscopic and bacteriological investigation may probably bring them more and more within the field of pathological research. Meanwhile we must regard a neurosis as a functional disorder of the nervous system, neurotic persons being prone to morbid manifestations of nerve action. Moreover, we must bear in mind that in some cases at least such manifestations depend not only upon inherent instability of nerve cells, but upon morbid conditions of the blood, by which these cells are bathed. Thus it is probable that both in epilepsy and migraine toxæmia is frequently the exciting cause of the attack; and the association of anæmia with hysterical and neurasthenic conditions is well-known. In hereditary neurosis there is probably a special susceptibility of the nervous structures to such influences; in other words, the nerve elements are unusually irritable. That a tendency to such irritability is transmitted by inheritance there is ample proof; and, as we shall see, the irritability shows itself in a variety of ways. The higher nervous centres are frequently involved, and intellectual instability may result. Mental disorder is, indeed (as Maudsley tells us), "neither more nor less than nervous disease, in which mental symptoms predominate."

We are all familiar with what are commonly designated nervous families; that is to say, families whose members are more liable than others to suffer from some neurosis. Amongst such we shall find children deficient in mental power or lacking in self-control; children prone to convulsions during teething, and at a later age to choreic affections; children who suffer from "nerve storms," which in some take the form of temper, in others of migraine, in others of epileptic attacks. The neurosis may even show itself as moral perversion, and may be known as moral imbecility or juvenile insanity.

The versatile character of neurotic inheritance is displayed in certain family histories that afford us the opportunities of tracing the characters of members of successive generations. The existence of neurotic tendency is not necessarily associated with mental inferiority. In some cases, indeed, it goes with talent of a certain kind. As Dryden long ago remarked—

> "Great wits to madness sure are near allied,
> And thin partitions do their bounds divide"; and it could easily be shown that youthful precocity, artistic ability, and even poetical genius are not uncommonly met with amongst persons of neurotic inheritance. The general history of a neurotic family is, however, not such as to justify the perpetuation of the strain; and we may quote from Nisbet's "Insanity of Genius" a typical example of the effect of such morbid taint. "An 'eccentric' marries a healthy woman, and has three sons and one daughter. The eldest son is sound, the daughter is weak-minded, the second son is also weak-minded, and the third is eccentric and ailing. The third son, who in spite of his ill-health lives to be 71, marries an intelligent and apparently sound woman, and has a large family, with the following characteristics: (1) Alexander, imbecile, lives to 69; (2) Ellen, sound and long-lived; (3) Mary, imbecile, dies at 12; (4) William, idiot, alive at 68; (5) John, idiot, dies at 56; (6) Robert, imbecile, dies at 61 (scrofulous); (7) Joan, consumptive, dies at 44, leaving a daughter, who also dies of consumption; (8) Thomas, a sufferer from chronic bronchitis with nervous exhaustion (*query, spasmodic asthma*), marries a sound woman, and has an imbecile son; (9) Anne, imbecile, dies at 44 of consumption; (10) James, well and long-lived, marries and has eight healthy children; (11) Charles, imbecile, dies at four." This melancholy pedigree shows how, from a single "eccentric" ancestor three-fourths of the progeny of the first generation, and nine-elevenths of the second, inherit some form of neurosis, for (as we shall see) there is reason to think that consumptive disease, in such a connection, is but a transformed neurosis. Unhappily there is no law, except that of prudence, to prevent members of neurotic families from intermarrying with other neurotics, and so propagating a degenerate race. The marriage, of which we have seen the calamitous consequences, was (we are told) between an "eccentric" man and a sound woman; and its prolific character may be explained by the latter factor. But at length a time comes when nature revolts against the procreation of the unfit; and degeneracy, intensified by imprudent marriages, tends to infertility, and the ultimate extinction of the race.

Morel (in his "Traité des Dégénérescences de l'Espèce Humaine"*) has traced backward through four generations the family history of a patient of his at the Rouen Asylum afflicted with mania at the age of sixteen, but apparently the subject of congenital defect. In the first generation (the boy's great grandfather) there was alcoholic excess and moral degradation; in the second, inherited alcoholism, maniacal attacks, and general paralysis; in the third, hypochondriasis, delusions of persecution, and homicidal tendencies. In the fourth (that of the patient) deficient intelligence, stupidity, dementia, and, finally, extinction of the race.

In this country, with a rapidly moving population, it is not easy to trace through successive generations the vicissitudes (viewed from a psycho-physiological standpoint) of a neurotic family. In Norway, where the population is more fixed to its own particular district, Dr. Dahl, in his book on insanity, gives a number of instructive pedigrees. These show not only the hereditary character of neurosis, but the variations of form assumed in different members of the same family. Thus we find insanity, idiocy, epilepsy, deaf-mutism, and even blindness alternating in the same neurotic stock.

* P. 123. "Traité des Dégénérescences, &c.," Paris, 1854.

